# Nanotechnology Interventions in the Management of COVID-19: Prevention, Diagnosis and Virus-Like Particle Vaccines

**DOI:** 10.3390/vaccines9101129

**Published:** 2021-10-04

**Authors:** Acharya Balkrishna, Vedpriya Arya, Akansha Rohela, Ashwani Kumar, Rachna Verma, Dinesh Kumar, Eugenie Nepovimova, Kamil Kuca, Naveen Thakur, Nikesh Thakur, Pankaj Kumar

**Affiliations:** 1 Patanjali Herbal Research Department, Patanjali Research Institute, Haridwar 249405, India; acharya.balkrishnapri@prft.in (A.B.); vedpriya.arya@prft.in (V.A.); akansha.rohela@prft.co.in (A.R.); 2 Department of Allied Sciences, University of Patanjali, Haridwar 249405, India; 3 School of Biological and Environmental Sciences, Shoolini University of Biotechnology and Management Sciences, Solan 173229, India; 4 School of Bioengineering and Food Technology, Shoolini University of Biotechnology and Management Sciences, Solan 173229, India; dineshkumar@shooliniuniversity.com; 5 Department of Chemistry, Faculty of Science, University of Hradec Kralove, 50003 Hradec Kralove, Czech Republic; eugenie.nepovimova@uhk.cz; 6 Biomedical Research Center, University Hospital in Hradec Kralove, Sokolska 581, 50005 Hradec Kralove, Czech Republic; 7 Department of Physics, Career Point University, Hamirpur 177001, India; naveen.phy@cpuh.in (N.T.); nikesh.phy@cpuh.edu.in (N.T.); h14862@cpuh.edu.in (P.K.)

**Keywords:** SARS-CoV-2, COVID-19, diagnosis, virus-like particle vaccines, prevention

## Abstract

SARS-CoV-2 claimed numerous lives and put nations on high alert. The lack of antiviral medications and the small number of approved vaccines, as well as the recurrence of adverse effects, necessitates the development of novel treatment ways to combat COVID-19. In this context, using databases such as PubMed, Google Scholar, and Science Direct, we gathered information about nanotechnology’s involvement in the prevention, diagnosis and virus-like particle vaccine development. This review revealed that various nanomaterials like gold, polymeric, graphene and poly amino ester with carboxyl group coated magnetic nanoparticles have been explored for the fast detection of SARS-CoV-2. Personal protective equipment fabricated with nanoparticles, such as gloves, masks, clothes, surfactants, and Ag, TiO_2_ based disinfectants played an essential role in halting COVID-19 transmission. Nanoparticles are used not only in vaccine delivery, such as lipid nanoparticles mediated transport of mRNA-based Pfizer and Moderna vaccines, but also in the development of vaccine as the virus-like particles elicit an immune response. There are now 18 virus-like particle vaccines in pre-clinical development, with one of them, developed by Novavax, reported being in phase 3 trials. Due to the probability of upcoming COVID-19 waves, and the rise of new diseases, the future relevance of virus-like particles is imperative. Furthermore, psychosocial variables linked to vaccine reluctance constitute a critical problem that must be addressed immediately to avert pandemic.

## 1. Introduction 

Virus epidemics have been increasing at an alarming rate in recent decades. In December 2019, the severe acute respiratory syndrome coronavirus 2 (SARS-CoV-2), or COVID-19, was reported in Wuhan, China, which caused a substantial amount of illness and mortality worldwide. With its exponential rise throughout China, it put the world in danger and became a pandemic [1,2,3,4,5]. SARS-CoV-2 was eventually proclaimed an International public health emergency by the WHO on 30 January 2020 [6]. COVID-19 had a significant impact on humans, nature, and the world economy, and it posed a severe threat to human life on a vast scale.

SARS-CoV-2 is disseminated via micro-droplets, which are emitted primarily from one person to another or by touching contaminated objects [7]. To stop COVID-19 from spreading, it is critical to use disinfectants and personal protective equipment (PPE) such as gloves and masks. It is necessary to minimize its spread and cure it using various methods, including isolation of infected patients, rapid detection technologies, and discovery of effective vaccines. The COVID-19 pandemic is unlikely to cease until vaccinations that protect against severe disease are widely available, and herd immunity is acquired [8]. Vaccines have been permitted or approved for human use in several countries, with more scheduled to be licensed before the end of 2021. The vaccination process is influenced by the willingness to vaccinate, which is part of the vaccine reluctance phenomenon. The World Health Organization has identified vaccine hesitancy as one of the most serious threats, urging researchers to investigate the variables that contribute to this problem. Because of low vaccination rates and a lack of an effective cure, governments have been forced to resort to social segregation and frequent lockdowns to combat COVID-19 [9]. On the other hand, the current treatments primarily provide symptomatic relief and are utilized to strengthen the respiratory system’s immunity to combat this virus [10]. In addition, some researchers are investigating the transmission similarities among SARS-CoV-2 and SARS-CoV to create treatments that target highly conserved essential proteins associated with viral replication and proliferation [11,12]. 

In this context, nanotechnology has a broad range of capabilities and opens up new opportunities to design novel preventive, diagnostic, and treatment approaches against COVID-19 and other viral infections. In pre-clinical tests, nanotechnology-based tools have been highly effective against many diseases, including respiratory viruses, herpes virus, human papillomavirus, and human immunodeficiency virus (HIV) [13,14]. Polymeric, inorganic, and organic nanoparticles (10^−9^) are biological agents, making them a promising tool [15,16,17,18,19]. Furthermore, a high surface to volume ratio, surface alteration properties, physicochemical stability, and specific optical characteristics all contribute to lower toxicity and greater efficiency, making nanoparticles (NPs) valid for the prevention, treatment, and diagnosis of viral infections such as COVID-19 [20,21,22]. Scientists have recently become interested in NP-based antiviral agents that utilize such NPs as gold, silver, titanium, iron, cadmium, and polymeric because of their encapsulation and optical characteristics for treating and diagnosing Ebola, HIV, influenza, and herpes simplex virus [23]. Subsequently, antiviral air filters coated with SiO_2_-Ag NPs, with a coating density > 2.0 × 10^8^ cm^2^, were claimed to prevent viral infection by 99.9% [24]. In hospitals, Ag NP-coated filters with TiO_2_ NPs were established as an enhanced air purification system [25]. Antiviral nanoparticles integrated in fabrics can help to overcome the problem of viral contamination in masks and PPE kits [26]. Interestingly, NPs have proven to be a promising tool in a variety of bio-sensing applications due to their customizable physiological features [27]. In addition, metal NPs, silica NPs, quantum dots (QDs), carbon nanotubes, and polymeric NPs have all been investigated in the context of viral detection [28,29]. Among them, metal NPs, metal nanoislands, magnetic NPs, and quantum dots have all been used to detect coronaviruses [30]. On the other hand, nanocarriers can facilitate the targeting and release of antigens or adjuvants to antigen-presenting cells [31,32]. Liposomes, carbon-based nanoparticles, polymeric nanocarriers, and emulsions have all been researched extensively, and they found applicability in the delivery of vaccines [33]. NPs including SiO_2_, TiO_2_, Bi_2_O_3_, Ag_2_O, FeO, MnO_2_, Al_2_O_3_, and others play important roles in a variety of medicinal applications [34,35]. In addition, AgS-, CuS-, FeS-, Zn-, and Cu-based metal–organic frameworks are frequently utilized in drug delivery [36]. NPs have been used as a drug delivery vehicle in several approved COVID-19 vaccines. 

In addition to this, virus-like particles (VLPs) could constitute an innovative vaccine approach to stop the pandemic. VLPs (nanoscale entities) are composed of integrated viral proteins that are non-infectious due to the absence of genetic material [37]. These structures mimic the size and shape of actual viruses and can effectively activate immune responses. VLPs are safer for immune-compromised or aged vaccine users as they lack viral genomes (non-replicating) [38]. VLPs’ uptake by antigen-presenting cells can result in effective immune responses, resulting in infection control, according to experience with VLP-based vaccinations [39]. VLPs are an excellent foundation for developing a safe and efficacious vaccine because of these characteristics [40]. Several groups are currently evaluating this technology as a SARS-CoV-2 vaccination approach [41]. Firstly, we provide a brief description of COVID-19 and the involvement of nanotechnology in its prevention and diagnosis. Secondly, in the context of COVID-19, existing vaccine approaches, vaccine statuses, futuristic insights into VLP vaccines, and COVID-19 vaccine hesitancy are highlighted. 

## 2. Search and Inclusion Criteria

A literature review was conducted between 2 May 2021 and 14 July 2021. Various keywords such as COVID-19, SARS-CoV-2, role of nanomaterials in prevention, diagnosis, treatment, vaccines against COVID-19, nanomaterials for biomedical application, virus-like particle, COVID-19 vaccine hesitancy, and unwillingness to receive vaccines were used in this review. Only papers indexed in Web of Science, and published in Scopus-indexed and peer-reviewed journals, were included in this study to maintain quality. 

## 3. COVID-19: A Brief Overview

COVID-19 emerged as an epidemic and has now infected millions of people globally, making it the most severe global danger of the new millennium [42]. SARS-CoV-2 is a Beta-coronavirus that belongs to the family *Coronaviridae*. SARS-CoV-2 is a single-stranded RNA virus with a 30 kb genome, and fourteen open reading frames encoding four structural proteins: nucleocapsid (N), spike (S), membrane (M), and envelope (E) [43,44,45]. Following China, several nations have been disproportionately affected, with the top ten being the United States, India, Brazil, Russia, France, Turkey, the United Kingdom, Argentina, Colombia, and Italy [46].

Beta-coronaviruses’ key characteristics are rapid mutation, varied tissue tropism, cross-species communication, and adaptation to various epidemiological circumstances [45,47,48,49]. According to the investigations, the COVID-19 virus’s causative agent shares 89, 82, and 96.3% nucleotide similarity with SARS-like CoV ZXC21, SARS-CoV, and bat CoV RaTG13, respectively [50,51]. Physical contact and fomites are the most prevalent routes for respiratory illnesses to spread. Virus transmission via physical contact is the direct transmission from an infected person to the next person, whereas fomites refer to the indirect transmission of the virus via intermediate objects [4]. The main symptoms of COVID-19 are cough, fever, and exhaustion, with shortness of breath, headache, anorexia, sore throat, and vomiting being less prevalent [52].

**Figure 1:**
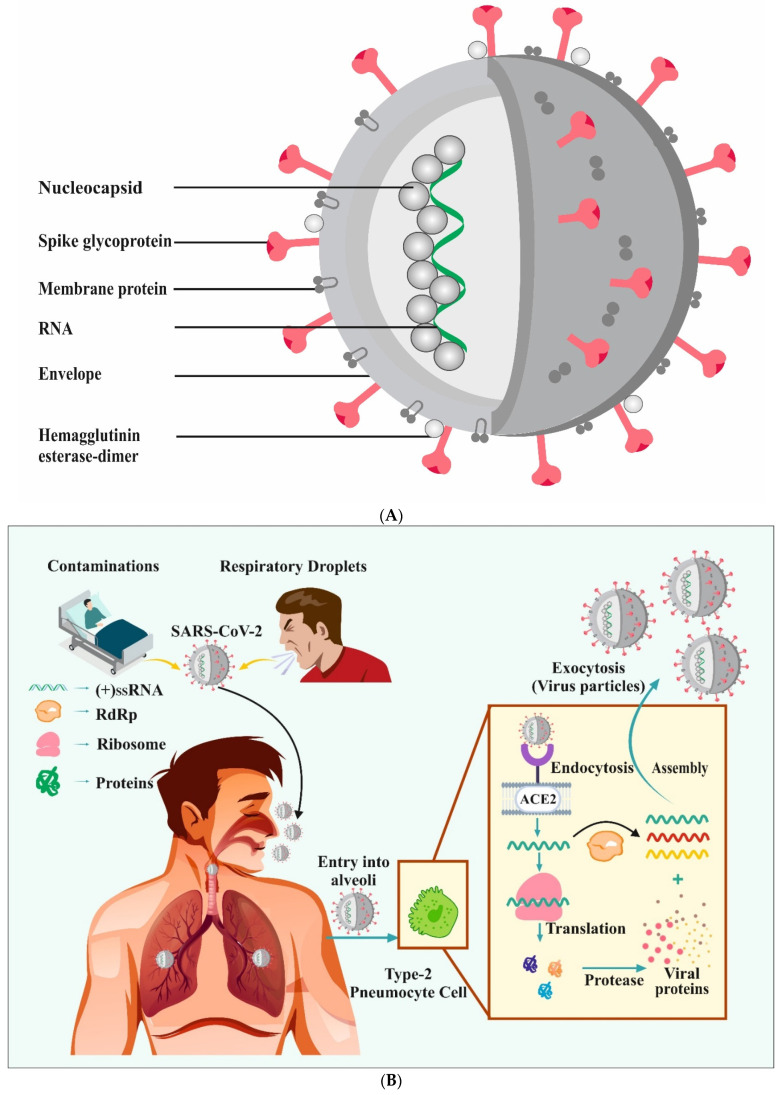
Schematic representation of SARS-CoV-2 structure (**A**). The life cycle of SARS-CoV-2 (**B**). The life cycle is reproduced from [51] under the Creative Commons Attribution (CC BY) license (http://creativecommons.org/licenses/by/4.0/) (accessed on 28 September 2021). RdRp: RNA-dependent RNA polymerase; ACE 2: angiotensin-converting enzyme-2 receptor.

A spike glycoprotein (S), a matrix protein (M), a nucleocapsid protein (N), and a small envelope protein (E), with sizes ranging from 60 to 110 nm, all contribute to the virus’s pathogenesis [53,54,55,56,57], as shown in Figure 1A. The N proteins, which are found in the endoplasmic reticulum-golgi area, combine with (+)ssRNA to produce nucleocapsid (helical) that aids viral reproduction [58]. The M protein also contains a prominent structure with three transmembrane domains related to the virus’s size, shape, and assembly [59]. The E protein is expressed within the infected cell’s vesicle trafficking organelles and elaborates at numerous stages for SARS-CoV-2 replication activities [60]. N and S proteins are particularly crucial as they are involved in viral infection and mediate the entrance of the virus into host cells [61,62]. Hemagglutinin esterase (HEs) is a glycoprotein that is found in some enveloped viruses and is used as an invasion mechanism. HEs aids in the attachment and degradation of specific sialic acid receptors found on the host cell’s surface [63].

The life cycle of SARS-CoV-2 is depicted in Figure 1B. Viral RNA enters the nucleus for replication when the viral components have penetrated the host cells, and viral mRNA is employed in the biosynthesis process to produce viral proteins. The viral S protein binds with the angiotensin-converting enzyme-2 receptor before entering the cells through endocytosis. Following entry, the virus envelope is proteolytically cleaved, releasing genomic RNA into the cytoplasm and producing smaller RNAs. The RNA-dependent RNA polymerase (RdRp) enzyme is essential for genome replication and transcription. The mRNAs are then translated to form several proteins that are essential for viral assembly, which, when entering the endoplasmic reticulum (ER), form nucleoprotein complex (a combination of N protein and genomic RNA). The entire virus particle is generated in the ER-Golgi apparatus area. After that, the viral particles are released by exocytosis [62,64,65].

## 4. Properties and Applications of Different Nanomaterials

**Figure 2:**
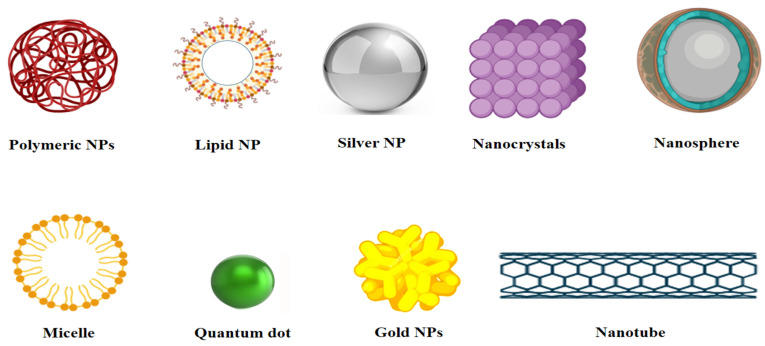
Different classes of nanoparticles. NPs: nanoparticles (created using biorender.com) (accessed on 28 September 2021).

NPs are divided into several categories of nanosystem based on their specific characteristics or features, such as inorganic, organic, lipid-based, polymeric, nanocapsules, nanospheres, virus-like particles, and others; some of them are illustrated in Figure 2. The excellent optical characteristics of inorganic NPs set them apart from other nanomaterials. Inorganic NPs have vital properties such as controlled stability, controlled release, improved permeability, and strong functionalization capability that lead to biomedical applications [66]. 

Other characteristics such as luminescence, size modifications, form, composition, and a high surface-to-volume ratio demonstrate adaptability in various therapeutic applications [67]. Mesoporous silica, metal oxide (FeO, TiO_2_, CuO, ZnO), and metallic NPs such as Au and Ag are the most prevalent nanomaterials among inorganic NPs. Furthermore, graphenes, carbon nanotubes (CNTs), and fullerenes NPs are organic NPs with exciting physical and chemical features for creative scientific and technological applications [68]. Organic NPs have high electric conductivity, allowing them to be used in various scientific domains [69]. Furthermore, solid lipid NPs, liposomes, nanoemulsions, and nanosuspension are all types of lipid-based NPs. They offer a wide range of therapeutic applications due to their increased surface area, controlled release, and improved drug delivery [70]. Antiviral medications such as maraviroc, ritonavir, zidovudine, efavirenz, lopinavir, and darunavir have been delivered using solid lipid NPs [71]. Nanoemulsions have a high water solubility, bioavailability, and lymphatic absorption, making them ideal for blending with such medications as saquinavir or indinavir [72].

Polymeric NPs are tiny particles with a diameter of 1 to 1000 nm that can entrap the active constituents that are within, or have been surface-adsorbed to, the polymeric core. Polymeric NPs have shown considerable promise in delivering medications to specific locations for the treatment of a variety of ailments [73]. Polymeric NPs have several advantages as drug carriers, including the capacity to control release, protect drugs and other biologically active compounds from the environment, and improve bioavailability and therapeutic indexes [74,75]. When used as a drug delivery carrier for HIV drugs (efavirenz, darunavir, or indinavir), polymeric micelles protect against degradation, in addition to improving solubility and taste in pediatric formulations [76]. Further, they are employed in carrying lamivudine stearate against Hepatitis B [77]. The polymeric NPs mediated delivery of nevirapine against HIV, increased therapeutic efficacy, and decreased biocompatibility [78]. Another type of nanomaterial is nanocapsules; they have a polymeric shell around an inner core and are utilized for targeted medication delivery. Nanocapsules, made up of a poly core with an azidothymidine triphosphate entrapment, are also reported for direct drug delivery to the cytoplasm [79]. According to a study, chitosan nanospheres containing acyclovir are more efficient than acyclovir alone in treating herpes [80]. On the other hand, nanospheres are smaller (10–200 nm) and are associated with rapid drug clearance. Importantly, VLP are composed of single or multiple viral entities that may self-assemble, and they are similar in shape and size to viruses but lack the genetic material to infect the host cell [81].

## 5. Role of Nanotechnology in Prevention and Diagnostic Approaches

In the realm of science, innovation is necessary. For example, with the rise of various diseases, nanostructures and nanotechnology-based products are consistently under dynamic development for novel preventive, diagnostic, and therapeutic approaches because of their affordability and toxicity. 

### 5.1. Nanotechnology in Prevention: A Brief Overview

The Centers for Disease Control and Prevention, United States, stated that significant influences for COVID-19 transmission by contact vary from person to person but involve the respiratory droplets of infected individuals [82]. To prevent the spread of the disease, the use of personal protective equipment (PPE) such as gloves and masks is essential. There are some limitations regarding the diversity of PPE, such as face masks that cannot prevent airborne viral particles [83,84]. Generally, the gaps between the fibers of facemasks range between 10 and 30 μm, and thus, they cannot prevent contact with the virus but cause breathlessness and increases in temperature and pressure [85]. NPs such as nanofibers can lower pressure and also diminish breathing resistance to provide comfort and protect against small particles of less than 40 nm [86]. Additionally, to curb the COVID-19 pandemic, a team of researchers at LIGC Applications Ltd., United States, manufactured a reusable mask composed of microporous conductive graphene foam that traps and kills microorganisms through the conduction of electrical charges [87]. In addition, researchers from the Queensland University of Technology, Australia, created a breathable filter cartridge made of cellulose nanofibers that could filter tiny particles (100 nm) [88]. Balagna et al. [89] revealed that silver nanocluster/silica composite-fabricated face masks inhibited SARS-CoV-2. In addition, Promethean Particles Ltd., in collaboration with textile companies, are currently exploring new possibilities for the use of copper NPs embedded in polymer fibers, through a melt extrusion process, in PPE for the protection of healthcare workers [90].

On the other hand, nanotechnology also finds applications in the development of effective antiviral surface disinfectants that can inactivate the virus and prevent its spread. Chemical disinfectants, amidst their positive results, are usually associated with significant drawbacks such as highly concentrated formulations for complete viral inhibition, constrained efficacy with time, and potential risks to society and the environment [1,91]. Due to their inherent antiviral potential, persistency, and efficacy at lower dosages, metallic NPs (copper, silver, titanium dioxide NPs, and others) can be used as an alternative solution [92,93]. For instance, a self-sterilizing solution was created by NanoTech Surface in Italy for sterilizing and disinfecting surfaces through the use of silver ions and titanium dioxide (TiO_2_) [90]. Similarly, a TiO_2_ NP-based photocatalytic coating was developed by FN Nano Inc. in the USA to decompose viruses present on the surface by damaging their viral membrane when exposed to light [90]. The nanomaterials have enormous potential as coronavirus disinfectants, owing to their unique characteristics, which include inherent antiviral qualities such as reactive oxygen species production, as well as photodynamic and photothermal capabilities. Furthermore, by employing biodegradable metallic NPs, the negative impacts of metallic NPs on public health and the environment may be avoided [94]. 

### 5.2. Nanotechnology-Based Diagnostic Approaches

In COVID-19-like pandemics, early diagnosis is critical for identifying cases and preventing infection. RT-PCR testing is the preferred method for the detection of COVID-19 in current instances. Antibody-based immunological tests, on the other hand, are easy-to-use procedures for quick screening. Even though there are numerous issues concerning the accuracy and sensitivity of fast detection kits, they are in high demand as they offer rapid diagnoses. The USFDA approved a large number of commercial diagnostic kits for COVID-19 diagnosis based on such methods as the molecular assay, the antigen test, the antibody-based manual test, and the automated immunoassay, and other tests that use molecular-based assays are susceptible (100%) [95].

Detailed information about various assays is available at https://www.finddx.org/covid-19/sarscov2-eval/ (accessed on 15 June 2021) [96]. The existing technologies have several drawbacks. For example, while the basic Q COVID-19 Ag test is quick and straightforward, it has a low positive predictive value in a low prevalence area. A negative test result can also arise if the amount of extracted antigen in a specimen is less than the test’s sensitivity or if the specimen is of poor quality [97,98]. Furthermore, a single RT-PCR test kit can cost more than $100, even though it requires 4–6 h of analysis time and, as a result, turnaround time of more than 24 h [99,100]. The RT-PCR test kit has a severe issue with high probabilities of false-negative COVID-19 diagnosis due to the high possibility of cross-contamination during sampling, dilution, and processing [101,102]. There is a strong need to establish more feasible, reliable, and accurate detection tests that provide faster findings in order to improve people’s quality of life.

In this regard, the utilization of nanomaterials has resulted in more sensitive, cost-effective, and suitable tools for diagnostic purposes [103,104,105,106,107]. Several nanomaterials such as quantum dots [108,109], carbon nanotubes, silica, graphene oxide, and metal NPs are often utilized in biosensors to detect viruses including herpes virus with influenza virus A, Kaposi’s sarcoma, human papillomavirus, hepatitis virus (A, B, E), Rift valley fever virus, *Hantaan orthohantavirus*, and HIV [110]. Therefore, in this review, we compiled a few studies representing the possible strategies against SARS-CoV and other viral strains, in addition to contemporary work against SARS-CoV-2 detection, which can be utilized to diagnose the latter. The gold NP-based immunochromatographic strip strategy seemed to possess the capability of on-ranch rapid identification of various infectious bronchitis virus (IBV) strains in chickens [111]. Moreover, a study performed by Teengam et al. [112] revealed that when using silver NPs, a colorimetric paper-based multiplex analytical instrument was fostered to detect DNA links that were accompanied by viral infections such as MERS-CoV, with a limit of detection of 1.53 nM. SARS-CoV nucleocapsid protein is a crucial antigen for the rapid detection of SARS infection. An on-chip approach was suggested by Roh and Jo [113] for the detection of the SARS-CoV N protein, which utilized a quantum dots-conjugated RNA aptamer with great sensitivity and speed. The limit of detection was found to be 0.1 pg/mL. 

**Figure 3:**
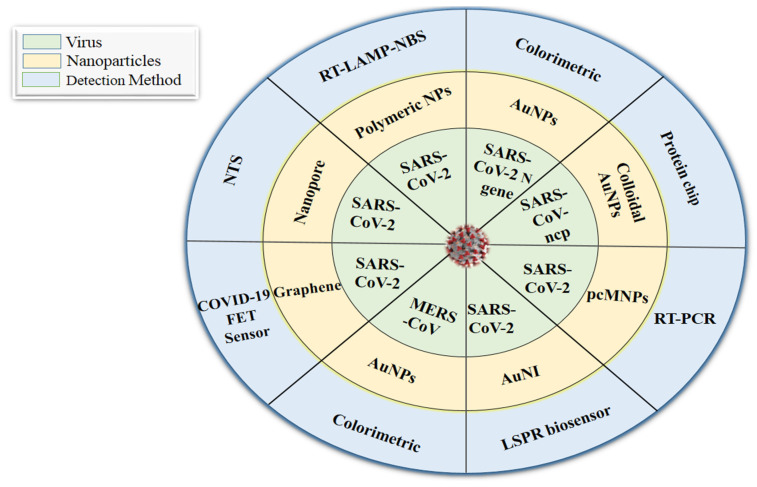
Nanotechnology-based approaches for MERS-CoV, SARS-CoV, and SARS-CoV-2 detection. Nanoparticles (NPs); gold NPs (AuNPs); field-effect transistor sensor (FET sensor); Middle East respiratory syndrome-coronavirus (MERS-CoV); severe acute respiratory syndrome coronavirus 2 (SARS-CoV-2); SARS-CoV-nucleocapsid protein (SARS-CoV-ncp); RT-LAMP associated with NP-based biosensor assay (RT-LAMP-NBS); Poly amino ester with carboxyl groups-coated magnetic NPs (pcMNPs); gold nanoislands (AuNIs); nanopore-targeted sequencing (NTS); localized surface plasmon resonance (LSPR); reverse transcription-polymerase chain reaction (RT-PCR).

The studies discussed above, which highlight the role of nanotechnology in viral detection, have encouraged researchers to develop rapid detection methods for COVID-19. The nanotechnology-based approaches for MERS-CoV, SARS-CoV, and SARS-CoV-2 detection are highlighted in Figure 3. Layqah and Eissa [114] outlined an electrochemical immunosensor to detect H-CoV (Human coronavirus) and MERS-CoV proteins in nasal samples by using a variety of carbon terminals that were modified with gold NPs (Au NPs), with a detection limit of 1 and 0.4 pg/mL, as well as linear ranges of 0.001–100 ng/mL and 0.01–10,000 ng/mL for MERS-CoV and H-CoV, respectively. Similarly, Xiang et al. [115] created point-of-care biosensors such as lateral flow assays coupled with NPs that were sensitive, fast, cheap, and simple to use against SARS-CoV-2. Likewise, Huang et al. [102] also developed Au NP-based lateral flow test to rapidly identify IgM antibodies against SARS-CoV-2. In addition to this, antibody-based biosensors are another possible way to detect COVID-19. For example, a new antibody-based biosensor was allegedly used to detect the SARS-CoV-2 spike protein. Patients’ nasopharyngeal swab samples were taken as antigens, and the SARS-CoV-2 antibodies were bound on graphene sheets of a field-effect transistor (FET). This sensor identified the virus with a detection limit of 1.6 × 10^1^ plaque-forming units (pfu)/mL. In addition, with a 2.42 × 10^2^ copies/mL detection limit, the COVID-19 FET sensor can distinguish between infected and healthy persons [116]. The colorimetric assay demonstrated in a study by Moitra et al. [117] was based on the capping of Au NPs with thiol-altered antisense oligonucleotides (ASOs) that are explicit for the N-gene (nucleocapsid phosphoprotein) of SARS-CoV-2; it exhibited a 0.18 ng/μL detection limit, and has been considered for utilization due to its capacity to diagnose COVID-19 instances within a few minutes using RNA samples. Additionally, Zhu et al. [118] established a single-step RT-LAMP (reverse transcription loop-mediated isothermal amplification) associated with a NP-based biosensor (NBS) assay (RT-LAMP-NBS) that was effectively used with 12 copies as a limit of detection.

Furthermore, another study described the development of a pcMNPs (poly amino ester with carboxyl groups-coated magnetic NPs)-based viral RNA extraction method for SARS-CoV-2 detection using RT-PCR. The limit of detection was reported to be ten copies [119]. The development of a dual-functional plasmonic biosensor that combined the plasmonic photothermal therapy with localized surface plasmon resonance (LSPR) sensing transduction was an alternative diagnosis option for COVID-19. The study was conducted through nucleic acid hybridization of gold nanoislands functionalized with cDNA receptors (complementary DNA) that may detect chosen sequences from SARS-CoV-2 with a 0.22 pmol/L detection limit [120]. Wang et al. [121] described the nanopore-targeted sequencing method for detecting SARS-CoV-2 and other respiratory viruses within 6–10 h, in this case, detection was limited to ten copies/reaction.

The various studies mentioned above suggest that nanoparticle-based instruments can be developed to diagnose various virus-linked diseases. As the COVID-19 pandemic is progressing rapidly throughout the world, improved nanotechnology diagnostic procedures should be used to halt the disease’s spread and accelerate the diagnosis. In addition, these technologies should be improved to be quickly applied in the event of an unexpected medical emergency.

Undoubtedly, the above studies provide a supportive approach corresponding to the successful application of nanostructures in the development of virus detection frameworks and treatment modalities, as well as prospective interventions in virus-like diseases such as COVID-19. Discovering nanotechnology-based methodologies to tackle COVID-19 will assist in conquering the constraints related to ordinary strategies for viral infection management [122]. 

## 6. Vaccines against COVID-19: The Role of Nanocarriers

Vaccination seems to be the most cost-effective approach in forestalling and combating irresistible pathogenic viruses such as SARS-CoV-2, which represent a global peril to human health. Currently, several organizations have made COVID-19 vaccines in less than a year, which is a remarkable accomplishment. In general, new vaccines usually take a decade or longer to foster [123,124,125,126]. According to a recent report, 289 Coronavirus vaccines are under development, with 66 of them in various phases of clinical testing, including twenty in phase three. Nevertheless, only five of the 66 vaccines have been approved, as of 3 February 2021, by stringent regulatory authorities or the World Health Organization (WHO). The five authorized vaccines were as follows: AstraZeneca in collaboration with Oxford University; BioNTech in collaboration with Pfizer; the Gamaleya Research Institute of Epidemiology and Microbiology, Russia; Moderna in collaboration with NIAID (the National Institute of Allergy and Infectious Diseases); and Sinopharm in collaboration with the Beijing Institute, China [8,41,127]. 

The AstraZeneca vaccine was reported to be a recombinant monovalent vaccine, made up of a single replication-deficient chimpanzee adenovirus (ChAdOx1) vector expressing SARS-S CoV-2′s glycoprotein, that requires a storage temperature of 2–8 °C [8,127,128]. In contrast, Ad26 (serotype 26) and Ad5 (serotype 5) are two recombinant replication-defective adenoviruses that were used in the Sputnik V vaccine, with a storage requirement of −18 °C. To induce an immunological response, the viruses were incorporated with the gene that produces the spike protein of SARS-CoV-2 [8,129,130]. Moderna, a mRNA-based vaccine encapsulated in lipid nanoparticles (LNP), requires a storage temperature of −20 °C [131,132], whereas the Sinopharm SARS-CoV-2 vaccine (Vero Cell) is an inactivated and adjuvanted (with aluminum hydroxide) vaccine that requires storage in between 2 and 8 °C to boost the response of the immune system [8,133]. Additionally, the WHO-approved vaccine by BioNTech in partnership with Pfizer is an mRNA-based vaccine encapsulated in a lipid nanoparticle that needs to be stored at −70 °C [7,8,119]. Furthermore, Janssen and Sinovac manufactured a non-replicating viral vector and an inactivated-virus vaccine, respectively, which recently passed the WHO vaccine evaluation process [8,19].

**Figure 4:**
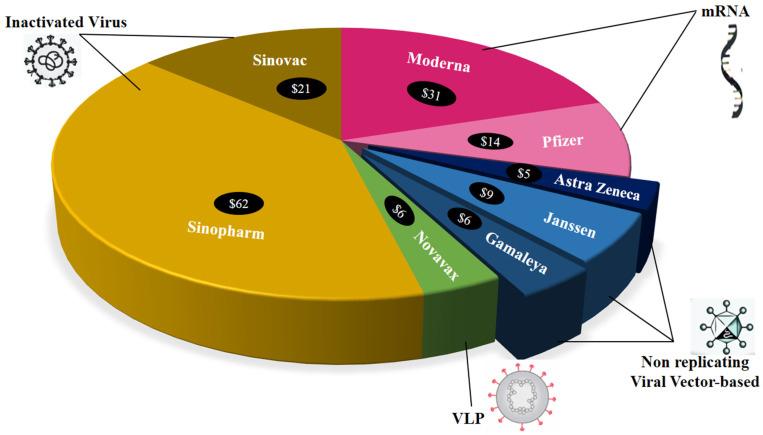
Affordability of some vaccines (these are the lowest pricing that the developers have ever provided to any country).

Importantly, nanocarrier systems can shield antigens from early degradation and offer prolonged release, improved antigen stability, and tailored immunogen delivery, as well as extending antigen exposition and uptake by antigen-presenting cells (APCs) [134,135]. Lipid NPs are employed for vaccine delivery as the nanocarrier system protects DNA or RNA from enzymatic destruction while increasing cell uptake and releasing the vaccine [136]. Similarly, there are other LNP-conjugated mRNA vaccines by Translate Bio/Sanofi Pasteur (Lexington/Bridgewater Township, U.S.); IMV, Inc. (Dartmouth, Canada); Fudan University/Shanghai JiaoTong University/RNACure Biopharma (Shanghai, China); CanSino Biologics/Precision NanoSystems (Tianjin, China/Vancouver, Canada); St. Petersburg Scientific Research Institute of Vaccines and Serums (Saint Petersburg, Russia) and many more, which were under pre-clinical development phase as reported by Campos et al. [7]. Some vaccine developers, such as AstraZeneca ($5 per course), Janssen by Johnson & Johnson ($9 per course), Gamaleya ($6 per course), and Novavax ($6 per course), committed to maintaining their low prices during the pandemic, whereas other vaccine developers, including Sinopharm ($62 per course), Sinovac ($21 per course), Moderna ($31 per course), and Pfizer ($14 per course), are charging considerably high amounts, as shown in Figure 4 [8].

Numerous vaccines have been licensed for human use against COVID-19, with many more in the final phases of clinical trials. However, having authorized vaccinations is not sufficient to accomplish global control of COVID-19: they additionally should be manufactured at a large scale, as well as being cost-effective and distributed internationally, so that they are accessible where they are required, and widely implemented in local communities [137]. Further, the current allocation choices are being made in the context of limited supply, with demand surpassing current and projected levels of output [138,139]. Shortage in supply combined with the enormous volumes of pre-orders made by more prosperous nations makes it challenging to accomplish vaccine access globally. In 2021, billions of people worldwide will probably not have access to coronavirus vaccines, which may extend the pandemic and increase the danger of new viral mutations, thereby jeopardizing the efficacy of existing authorized vaccines. In addition, re-infection and mutation in SARS-CoV-2 strains are other critical aspects affecting the population. Thereby, there is a need for a single effective novel vaccine that will protect individuals from various mutated SARS-CoV-2 strains and re-infection as well as enhance drug target delivery.

## 7. Virus-Like Particle (VLP) Vaccines 

**Figure 5:**
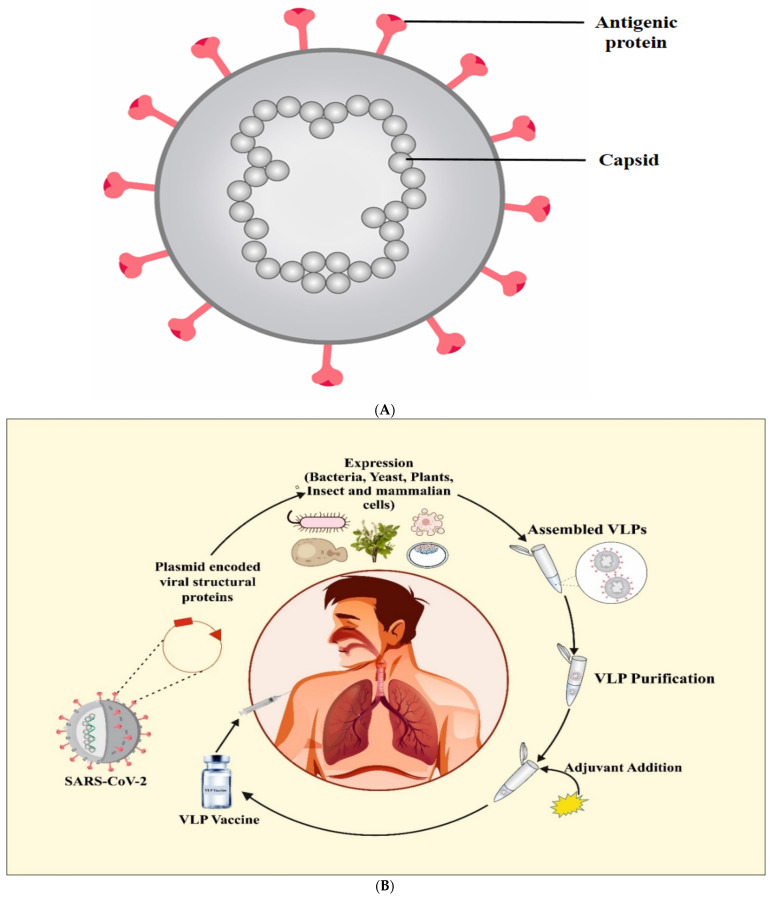
Virus-like particle (**A**). Virus-like particle vaccine development using various expression systems (**B**).

Several labs are now testing the virus-like-particles platform as a SARS-CoV-2 vaccination approach [41]. VLPs are non-infectious (no viral genome), antigenic nanostructures made from self-assembled viral proteins, as shown in Figure 5A [37,140,141]. These nanoparticle formations were initially discovered in the sera of Down’s syndrome, hepatitis, and leukemia patients in 1968. Interestingly, antigenic sites on their surface were also discovered [142]. VLPs are highly organized structures that are readily identifiable by immune system cells and molecules [143,144]. Experimentally, VLPs are made by utilizing viral proteins that are produced in various expression systems such as prokaryotic cells [145], yeast [146], insect cell lines [147,148], plants [149], and mammalian cell lines [143,150], as depicted in Figure 5B. Cloning of the viral structural genes and expression of viral proteins with self-assembling capacity in an appropriate expression platform are the first steps in the manufacturing process for VLP-based vaccines, as detailed above. After that, the assembled VLPs are subjected to downstream processing to obtain the purified intact VLPs. Adjuvants and other components are added in the following processes to develop a vaccine that is safe, affordable, and efficacious (Figure 5B) [81]. Usually, most VLPs are made from a single virus’s protein(s), but chimeric VLPs can be made by combining structural proteins from distinct viruses [145]. VLPs have been created using structural proteins from viruses such as HIV, adeno-associated virus, Hepatitis B, C, and bacteriophages [148,149,150]. 

Interestingly, VLPs are characterized as enveloped and non-enveloped, based on the lipid envelope’s presence or absence or on the basis of the arrangement of proteins into single, two, or multi-layered structures [151]. VLPs can be employed as nanocarriers as they have an interior cavity, and can be utilized to convey a variety of biological materials, including peptides, proteins, and micro drugs [152,153,154]. Besides this, they also offer numerous advantages over the existing vaccination platforms such as subunit (protein or polysaccharide), viral (live-attenuated or inactivated), nucleic acid (DNA or RNA), and viral vector vaccines. For example, VLP-based vaccines have been exploited as an alternative to attenuated or inactivated viruses because they avoid complete inactivation and inversion of viruses. Moreover, viral vector vaccines possess a risk of genomic integration. Additionally, nucleic acid vaccines such as DNA vaccines are difficult to administer, while low temperatures are required for RNA vaccinations, and there is a possibility of an RNA-induced interferon response [155,156]. Interestingly, it has also been observed that VLPs are easily absorbed and identified by antigen-presenting cells (APCs) due to their ideal size (20 to 200 nm) and particulate structure. Moreover, they provide high-density B-cell epitopes for antibody formation and intrinsic T-cell epitopes that induce strong humoral and cellular immunological responses, respectively [81,141,157]. 

Undoubtedly, VLPs are acquiring prominence as a preventive care. VLP-based potential vaccines have been licensed and marketed for human use against human papillomavirus (Cervarix^TM^ and Gardasil^®^) and for clinical use against hepatitis B virus (Engerix^®^ and Recombivax HB^®^) [158]. Besides this, Lokugamage et al. [159] also found that chimeric VLPs containing SARS-CoV S protein and mouse hepatitis virus M, E, and N proteins may develop neutralizing antibodies and lower the SARS-CoV titer in lungs of mice. Subsequently, Liu et al. [160] showed that chimeric VLPs made up of SARS-CoV S protein and influenza virus M1 protein could induce antibodies and protect mice. VBI vaccines Inc. is testing a multivalent eVLP vaccine that includes antigenic protein molecules of SARS-CoV-2, SARS-CoV, and MERS-CoV on a single particle. It is also called trivalent because it is made up of three distinct protein components. It is advantageous because it enables the formation of broad-reactive antibodies, which guard against SARS-CoV-2 strains that may become mutated over time [2,161]. The most contemporary VLP-vaccine against COVID-19 is Novavax, Inc. (United States), the efficacy of which is being screened in phase 3 trials. It contains SARSCoV-2 S protein integrated with an adjuvant matrix [7,8]. Besides this, there are 18 VLP-based vaccine candidates under pre-clinical trial against SARS-CoV-2, while five are at the clinical phase, as reported by the WHO report of July 2021.

## 8. COVID-19 Vaccine Hesitancy: A Major Concern 

COVID-19 vaccine acceptance is critical for achieving substantial immunization to eradicate the global pandemic. Large variability in COVID-19 vaccination acceptability rates has been reported across the globe. The vaccine acceptability is influenced by cognitive, psychologic, socio-demographic, and cultural factors [162,163]. An investigation of the aforementioned components is required to address COVID-19 vaccine hesitancy. As a result, vaccine reluctance has been the subject of numerous studies undertaken throughout the world [162,164]. Ecuador, Malaysia, Indonesia, and China had 97, 94.3, 93.3, and 91.3% COVID-19 vaccination acceptance among adults. In the Middle East, Africa, Russia, and various European nations, low rates of COVID-19 vaccine adoption have been recorded [165]. Ebrahimi et al. [166] investigated the psychological, socio-demographic, and contextual factors concerning vaccination hesitancy in 4571 Norwegian adults. Males, rural inhabitants, and parents with children under the age of 18 were identified as subgroups who were unwilling to receive vaccination. There were no distinctions in terms of education or age groupings. Subsequently, a survey of 788 adults in the United States was conducted to look into the relationships between demographics and psychosocial determinants of intent to receive a COVID-19 vaccine under emergency use authorization (EUA). Among them, 22.3% said they were definitely not willing. Individuals with a bachelor’s degree or above, men, and those who are insured expressed an interest in receiving vaccination against COVID-19 [167]. 

Solís Arce et al. [168] analyzed COVID-19 vaccine acceptability in 15 survey samples with 44,260 people from 10 low and middle-income countries (LMICs) in Asia, Africa, South America, Russia, and the United States. LMIC samples have a much higher willingness (80.3%) than the US (64.6%) and Russia (30.4%). In LMICs, vaccine uptake is generally explained by a desire for personal protection, with side effects being the most common cause for hesitancy. A cross-sectional study conducted by Xiao et al. [169] revealed that 1411 Chinese respondents were willing to receive the COVID-19 vaccine (N = 2528). Moreover, after the EUA, people’s willingness to receive vaccination was mostly impacted by coping appraisals rather than threat appraisals. On the other hand, Eastern Europe, the Middle East, and Russia had the lowest COVID-19 vaccination adoption rates. High adoption in East and Southeast Asia would aid in the pandemic’s effective management. In addition to Central and South America, more research is needed to investigate the attitudes of the individuals in Central Asia, Africa, and the Middle East. Such research would aid in assessing COVID-19 vaccination hesitancy and its subsequent risks in these areas, as well as around the world.

## 9. Conclusions and Future Perspectives 

In conclusion, the severity of COVID-19 has underlined the importance of innovative technical approaches for restricting and halting the disease. To begin with, nanoparticle-based products (disinfectants, NPs-fabricated PPE kit) aid in the prevention of COVID-19 transmission. The safe administration of Pfizer and Moderna vaccines using lipid NPs was found to be effective in controlling the pandemic in a number of nations. Researchers from all over the world have developed nano-based rapid detection methods that can be used in the near future if subsequent COVID-19 waves appear. VLP vaccines are under development. One developed by Novavax is awaiting WHO approval; if approved, a safer vaccine will be available, as VLPs are devoid of genetic material. Even though there is confirmation of safety and efficacy, very few vaccines are licensed and utilized in a broad population, so safety assessment of extremely modern technologies such as DNA, RNA, and VLPs should be given greater priority. Although NPs can be advantageous in biomedical applications, they also have a negative side, particularly toxicity, which must be considered carefully in order to maximize their usage in COVID-19 treatment. Finally, vaccine reluctance reports will aid in the development of educational materials and initiatives to encourage vaccination. Ongoing studies to address these issues must continue. 

## Data Availability

Not applicable.
